# Caring for refractive error equipment

**Published:** 2024-05-15

**Authors:** Ismael Cordero

**Affiliations:** 1 Clinical Engineer, Philadelphia, USA.

**The success of a community refractive error service depends on having well- maintained equipment that is in good working condition.**

**Figure:**
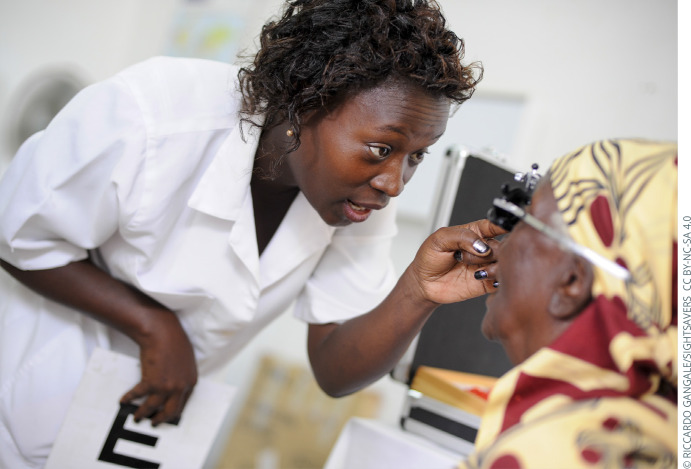
Refractive error examination. mozambique

In this article, we present a list of the equipment commonly used to provide refractive error services in lower-resource settings, along with guidance on care and maintenance. Where available, we have added links to previous articles in the *Community Eye Health Journal*.

**Distance visual acuity charts.** These charts have lines of optotypes (characters) of various sizes and are used to determine visual acuity at different distances. The Snellen chart displays letters or symbols at a standardised distance (usually 20 feet or 6 metres). Visual acuity is measured based on the smallest line a patient can read accurately at that distance. For patients who have difficulty recognising letters, a tumbling E chart may be used; this uses the letter ‘E’ in various orientations. Distance visual acuity charts are used during refraction to establish the best corrected distance visual acuity.

**Near visual acuity charts.** Near vision charts can consist of paragraphs of text with different font sizes, or they can consist of lines of numbers or tumbling Es in different sizes. These are used to check near vision at a normal near working distance for the patient (typically 40 cm).

**Tape measure.** Used to measure the distance between the patient and the near eye charts. This can help demonstrate to patients the closer working distance required for high positive lenses that may be needed to read ore perform close tasks at near.

**Occluder.** An occluder is used to cover one eye while the other is being tested.

**Pinhole.** A pinhole provides a simple way to focus light temporarily removing the effects of refractive error such as myopia.

**Cross cylinders.** Used to refine astigmatic prescriptions subjectively. The Jackson Cross Cylinder (JCC) consists of two cylinders at right angles to each other; by rotating the cylinder and observing the patient's responses to different lens orientations, the optometrist can determine the precise cylindrical power and axis needed to correct the astigmatism present.

**Trial lens set and trial frames.** A trial lens set contains lenses of different powers which can be temporarily placed in a trial frame (Figure 1). This enables the practitioner to determine the appropriate prescription for glasses using both objective and subjective methods. Initially, the lenses are used to accurately and objectively estimate the amount of refractive error present. The trial lenses are then used to subjectively refine the refractive error. **Trial frames** hold the trial lenses over each eye. For more information on their function and care, read our article, ‘**Understanding and looking after a retinoscope and trial lens set**’ (tinyurl.com/CEHJ-retino).

**Figure 1:**
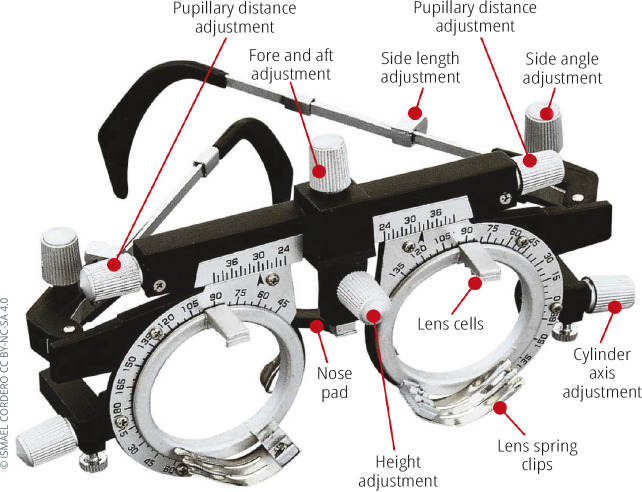
Components of a trial frame

**Pupillary distance ruler.** Used to measure the distance between the pupils.

**Lensmeter.** Also known as lensometer or focimeter, it is used to measure the prescription of a pair of eyeglasses. For more information read our article, ‘**Understanding and caring for a lensmeter**’ (tinyurl.com/CEHJ-lensmeter).

**Ready-made spectacles.** These pre-made glasses have the same powered lens on each side and can be dispensed immediately. These are useful where limited resources do not permit custom-made spectacles.

**Retinoscope** (Figure 2). This is a handheld instrument which shines a light into the eye – the reflection is observed and used to assess the amount of refractive error. It provides an objective method of refraction in which the patient does not need to tell the practitioner what they see. For more information, read our article, ‘**Understanding and looking after a retinoscope and trial lens set**’ (tinyurl.com/CEHJ-retino).

**Figure 2:**
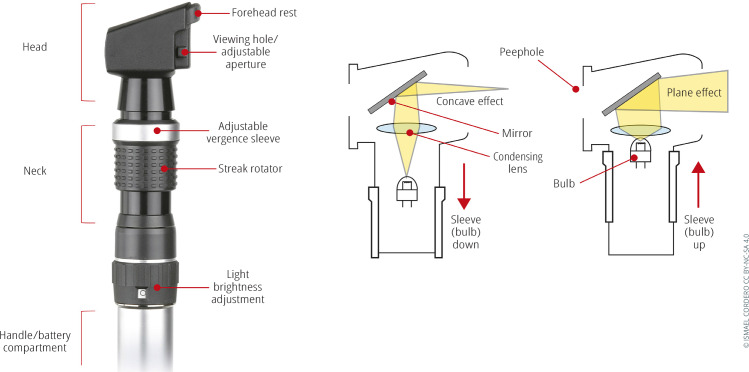
Components of a streak retinoscope

**Autorefractor.** A computerised device which can be used to objectively estimate a person's refractive error. Care needs to be taken when using these devices, particularly with children, as the accuracy of the results can be affected by accommodation.

## General care and maintenance

To ensure the proper operation, and to get the longest life from your refractive error devices, there are some essential practices that you should follow.

1. **General cleaning**
  - Some surfaces may be damaged by harsh cleaners and alcohol. Only use the cleaning solutions recommended by the manufacturer.
  - Keep instruments covered when not in use.
2. **Battery maintenance.** Batteries should be managed to ensure that medical devices are always ready for use. Here are a few tips:
  - Follow manufacturer guidelines.
  - Store the battery separately from the device if is not being used for a long time.
  - Keep an inventory of spare batteries.
3. **Cleaning lenses and optical surfaces.** Many ophthalmic devices have optical components such as windows, lenses, mirrors, filters, and prisms; these need to be protected and cleaned. For more information read our article, ‘**How to care for and clean optical surfaces’** (tinyurl.com/CEHJ-cleanopt).
4. **Prevention of fungal growth on optical components.** In hot and humid climates, it is common for mould to grow on the surfaces of optical components. Precautions should be taken to prevent this. For more information read our article, ‘**Fungus: how to prevent growth and remove it from optical instruments**’ (tinyurl.com/CEHJ-fungus).
5. **Bulb maintenance.** Many devices used in eye care rely on light bulbs or lamps for their operation. All light bulbs have a limited lifespan, and when the bulb fails the device becomes unusable. Therefore, knowing how to handle, inspect, and replace bulbs is important. Always maintain a supply of replacement bulbs for your equipment and only use those recommended by the manufacturer. For more information read our article, ‘**How to handle and care for bulbs in ophthalmic equipment**’ (tinyurl.com/CEHJ-bulbs).
6. **Fuse maintenance.** Fuses play an important safety role in preventing damage to equipment due to electrical overloading and reducing the risk of electrical shock to patients and staff. For more information read our article, ‘**Checking and replacing fuses**’ (tinyurl.com/CEHJ-fuses).
7. **General electrical safety practices.** For more information read our article, ‘**Electrical safety in the clinical environment – good habits to maintain**’ (tinyurl.com/CEHJ-electric).

